# Supplementary material for: Constipation Mitigation by Rhubarb Extract in Middle-Aged Adults Is Linked to Gut Microbiome Modulation: A Double-Blind Randomized Placebo-Controlled Trial
Source: Int J Mol Sci. 2022 Nov 24;23(23):14685. doi: 10.3390/ijms232314685 (PMC9738964; doi:10.3390/ijms232314685)
Supplement: Supplementary file 1 [file ijms-23-14685-s001.zip › ijms-1985807-supplementary.pdf]

Supplementary Materials

Title: Constipation mitigation by rhubarb extract in middle-aged adults is linked to gut microbiome modulation: a double-blind randomized placebo-controlled trial

Authors: Audrey M. Neyrinck, Julie Rodriguez, Bernard Taminiau, Florent Herpin, Patrice D. Cani, Georges Daube, Laure B. Bindels and Nathalie M. Delzenne

Table S1. Percentages of subjects that increased (↗), did not change (=) or decreased (↘) their consumption of food products in each group.

	Placebo (n=13)			REx1 (n=13)			REx2 (n=13)		
	↗	=	↘	↗	=	↘	↗	=	↘
**Bread, rusks or breakfast cereals**	23.1%	38.5%	38.5%	15.4%	61.5%	23.1%	61.5%	30.8%	7.7%
**Rice, pasta, potatoes or semolina**	30.8%	53.8%	15.4%	23.1%	69.2%	7.7%	15.4%	61.5%	23.1%
**Legumes**	38.5%	46.2%	15.4%	23.1%	53.8%	23.1%	30.8%	30.8%	38.5%
**Dairy products**	30.8%	30.8%	38.5%	38.5%	53.8%	7.7%	30.8%	38.5%	30.8%
**Fruit (including fruit juice)**	53.8%	30.8%	15.4%	46.2%	30.8%	23.1%	30.8%	38.5%	30.8%
**Vegetables**	61.5%	15.4%	23.1%	23.1%	53.8%	23.1%	30.8%	23.1%	46.2%
**Meat, poultry and eggs**	7.7%	46.2%	46.2%	15.4%	76.9%	7.7%	15.4%	53.8%	30.8%
**Fish and other seafood**	23.1%	53.8%	23.1%	30.8%	30.8%	38.5%	15.4%	69.2%	15.4%
**Total added fat**	7.7%	92.3%	0%	7.7%	84.6%	7.7%	0%	84.6%	15.4%
**Sweetened foods**	23.1%	61.5%	15.4%	23.1%	76.9%	0%	7.7%	84.6%	7.7%
**Salt**	0%	100%	0%	15.4%	84.6%	0%	7.7%	76.9%	15.4%
**Beverages (water *versus* other beverages)**	15.4%	69.2%	15.4%	23.1%	69.2%	7.7%	30.8%	38.5%	30.8%

Fisher test ($p > 0.05$)

Table S2. Number (N) and proportion (%) of subjects suffering or not of symptoms accompanying abdominal pain or discomfort the last 15 days in middle-age adults receiving rhubarb extract or placebo for 30 days[1].

		**Placebo**		**REx1**		**REx2**	
		N	%	N	%	N	%
**Abdominal pain or discomfort with diarrhea or loose stools**							
Day -14	No	13	100.0	13	100.0	12	92.3
	Yes	0	0	0	0	1	7.7
Day 30	No	13	100.0	11	84.6	8	61.5
	Yes	0	0	2	15.4	5	38.5
McNemar test		p>0.05		p>0.05		*p<0.05	
**Abdominal pain or discomfort with bloating**							
Day -14	No	7	53.8	9	69.2	6	46.2
	Yes	6	46.2	4	30.8	7	53.8
Day 30	No	10	76.9	10	76.9	11	84.6
	Yes	3	23.1	3	23.1	2	15.4
McNemar test		p>0.05		p>0.05		p>0.05	
**Abdominal pain or discomfort with gas**							
Day -14	No	7	53.8	9	69.2	7	53.8
	Yes	6	46.2	4	30.8	6	46.2
Day 30	No	10	76.9	10	76.9	10	76.9
	Yes	3	23.1	3	23.1	3	23.1
McNemar test		p>0.05		p>0.05		p>0.05	

Table S3. Mental and physical wellbeing according to Short-Form 12-item (SF-12) questionnaire in middle-aged adults receiving placebo or rhubarb extract at 2 doses for 30 days[1].

	**Placebo**		**REx1**		**REx2**	
	Day -14	Day 30	Day -14	Day 30	Day -14	Day 30
Mental Component Score (MCS)	45.6 ± 6.1	49.3 ± 6.0	46.5 ± 6.1	49.6 ± 8.4	46.4 ± 8.6	48.0 ± 7.2
Physical Component Score (PCS)	49.6 ± 6.8	52.8 ± 4.8	54.0 ± 5.9	49.6 ± 6.4*	52.0 ± 3.9	53.4 ± 4.1
Physical function	82.7 ± 21.4	92.3 ± 15.8	94.2 ± 11	86.5 ± 16.5	92.3 ± 12.0	98.1 ± 7.0
Role physical	83.7 ± 14.8	89.4 ± 16	89.4 ± 12.3	82.7 ± 16.6	83.7 ± 14.8	90.4 ± 11.6
Bodily pain	73.1 ± 27.9	92.3 ± 15.8*	90.4 ± 16.3	82.7 ± 18.8	80.8 ± 18.1	84.6 ± 21.7
General health	65 ± 12.6	66.9 ± 13.6	66.9 ± 13.6	63.1 ± 15.8	66.9 ± 13.6	72.7 ± 14.8
Vitality	32.7 ± 15.8	38.5 ± 19.4	38.5 ± 24.2	34.6 ± 26.1	38.5 ± 26.2	34.6 ± 28.0
Social function	84.6 ± 16.3	96.2 ± 9.4	96.2 ± 9.4	96.2 ± 13.9	92.3 ± 12.0	90.4 ± 12.7
Role emotional	80.8 ± 15.0	91.4 ± 11.8*	84.6 ± 10.4	91.4 ± 10.7	80.8 ± 27.3	91.4 ± 14.8
Mental health	64.4 ± 16.8	71.2 ± 12.9	65.4 ± 11.6	69.2 ± 20.8	66.4 ± 17.2	71.2 ± 16.4

[1]Values are means ± SD (n = 13). Matched-pairs Wilcoxon signed-rank tests were performed to compare changes from day -14 (within-group variations; *p < 0.05).

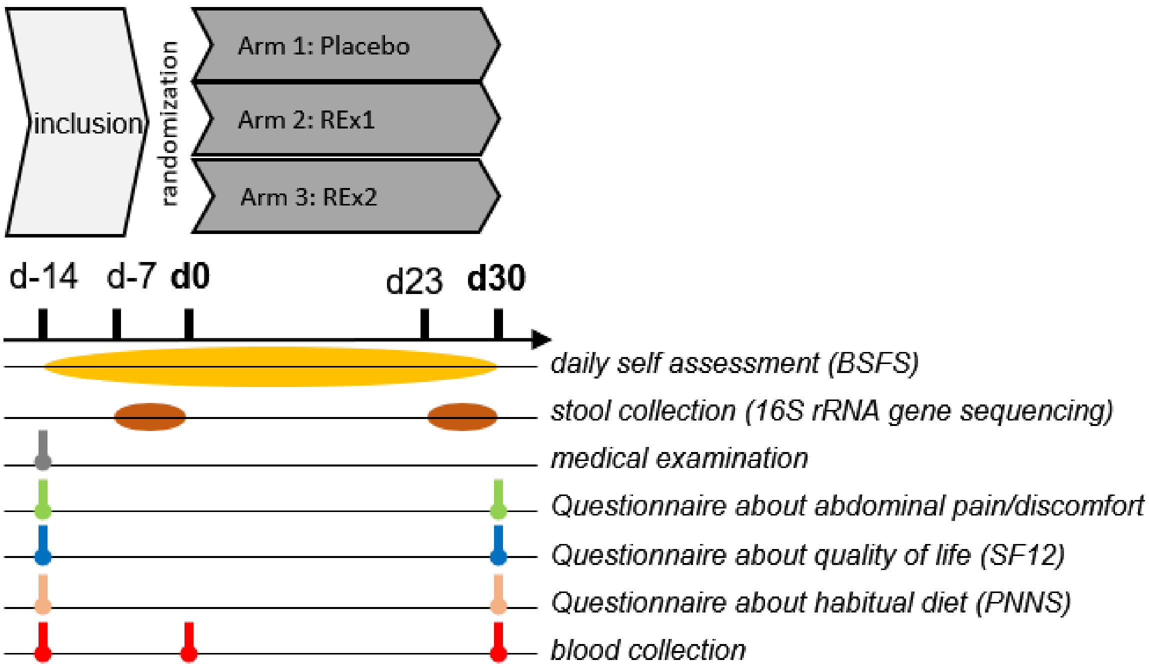

BSFS Bristol Stool Form Scale; SF12, 12-item short form survey measuring the eight health domains for adults (providing psychometrically based physical component summary and mental component summary scores), PNNS French National Nutrition and Health Program.

Figure S1. Overview of the clinical study

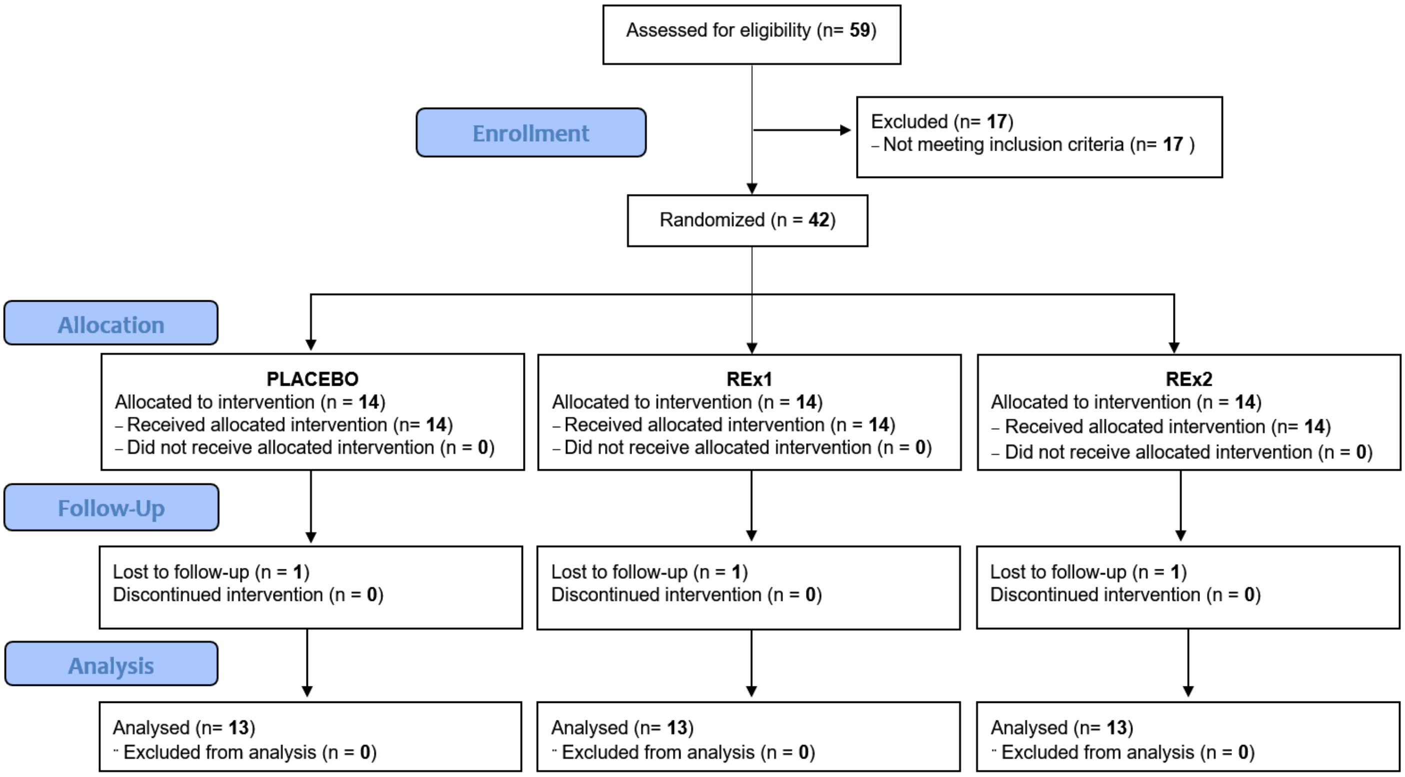

Enrollment and randomization of subjects according to the CONSORT Flow Diagram.

Figure S2. CONSORT Flow Diagram

Supplementary Methods

The inclusion criteria included:

- age between 50 to 70;
- subjects with ≥ 1 and ≤ 3 bowel movements per week in the month before the selection visit and two weeks before the enrolment visit
- subjects who met ROME III criteria for constipation [44], meaning that the participant reported experiencing at least 25% of defecations with two or more of constipation-related symptoms and experiencing rare loose stools without laxative use (i.e. less than three bowel movements per week; hard or lumpy stools more than 25% of the time, according to the Bristol board scale) at least the last 3 months over a period of 6 months;
- not justifying at the time of inclusion of a medical treatment according to his / her doctor;
- Accept and sign the consent

The exclusion criteria included:

- signs requiring further investigation: odynophagia, involuntary weight loss> 10% during the last 3 months before inclusion, persistent vomiting, hematemesis, blood in the stool, iron deficiency anemia, obstruction symptoms, rectal bleeding, rectal prolapse...;
- subject who has not been screened for colon cancer in the past two years;
- subject participating in another therapeutic trial;
- subject presenting a serious general pathology and in particular a renal or hepatic insufficiency, cancer, chronic pancreatitis;
- non-menopausal woman;
- subject with known hypersensitivity to one of the constituents of the product under study;
- subject who does not have the legal or ethical capacity to contract because of an impairment of cognitive function;
- subject who may not be compliant with the constraints imposed by the protocol;
- subject not benefiting from a health insurance plan.
- subject who, in the 30 days preceding the selection visit or who is currently taking medication, supplements and any food enriched or presented as containing substances, bacteria or yeasts likely to have an effect on the intestines and more particularly on

intestinal transit, digestive comfort, gas production, the occurrence of abdominal pain. (These products will also be banned for the duration of the study);

- subject following a particular diet (vegetarian, vegan, hyper-protein, ...);
- subject to a low caloric diet and followed by a doctor or dietician current or recent (<6 weeks);
- subject following medical treatment which, according to the investigator, could interfere with the evaluation of the study criteria: antibiotic, corticosteroid, anticholinergic, antidepressant, antiemetic, antihistamine, diuretic, calcium antagonist, antiparkinsonian, antipsychotic, antacid, analgesic, NSAIDs, H2-receptor antagonist, hypnotic, sedative, iron supplement, opioid and narcotic, laxative, anti-diarrheal, anti-reflux;
- subject having an alcohol consumption of more than 3 glasses of wine a day, or two glasses of beer a day, or a glass of hard liquor a day;
- subject having a coffee consumption greater than 5 cups per day;
- smoking subject;
- subject with a BMI higher than 30;
- subject with constipation attributable to an organic or anatomical cause (Hirschsprung's disease, hypothyroidism, mental deficiency, psychiatric illness, neurological abnormalities, history of operation of the colon or anus, colorectal cancer, anemia, etc.;
- subject with severe constipation (less than 1 stool per week during the 15 days preceding the inclusion visit);
- subject having a fiber intake higher than the recommended intakes (more than 6 fruits and vegetables per day according to the PNNS questionnaire);
- subject with pelvic floor dysfunction;
- subject with type 1 or type 2 diabetes;
- subject with a history of current gastrointestinal pathology or disorder such as duodenal ulcer, chronic colitis or chronic inflammatory disease of the gastrointestinal tract (Crohn's disease, ulcerative colitis), celiac disease or syndrome irritable bowel;
- subject having a history of operation of the digestive tract;
- subject having undergone surgery in the two months preceding the study;
- subject having undergone bariatric surgery;
- subject having a practice of intense sport activity (more than 10 hours per week of intense activity as defined by WHO).
